# Endothelial cell Notch signaling programs cancer-associated fibroblasts to promote tumor immune evasion

**DOI:** 10.21203/rs.3.rs-4538031/v1

**Published:** 2024-06-11

**Authors:** Yu Zhu, Menglan Xiang, Kevin F. Brulois, Nicole H. Lazarus, Junliang Pan, Eugene C. Butcher

**Affiliations:** 1 Laboratory of Immunology and Vascular Biology, Department of Pathology, Stanford University School of Medicine, Stanford, CA, USA.; 2 Palo Alto Veterans Institute for Research, Veterans Affairs Palo Alto Health Care System, Palo Alto, CA, USA.

## Abstract

Stromal cells within the tumor tissue promote immune evasion as a critical strategy for cancer development and progression, but the underlying mechanisms remain poorly understood. In this study, we explore the role of endothelial cells (ECs) in the regulation of the immunosuppressive tumor microenvironment. Using mouse pancreatic ductal adenocarcinoma (PDAC) models, we found that canonical Notch signaling in endothelial cells suppresses the recruitment of antitumor T cells and promotes tumor progression by inhibiting the pro-inflammatory functions of cancer-associated fibroblasts (CAFs). Abrogation of endothelial Notch signaling modulates EC-derived angiocrine factors to enhance the pro-inflammatory activities of CAFs, which drive CXCL9/10-CXCR3-mediated T cell recruitment to inhibit tumor growth. Additionally, abrogation of endothelial Notch unleashed interferon gamma responses in the tumor microenvironment, upregulated PDL1 expression on tumor cells, and sensitized PDAC to PD1-based immunotherapy. Collectively, these data uncover a pivotal role of endothelial cells in shaping the immunosuppressive microenvironment, and suggest the potential of targeting EC-CAF interaction as a novel therapeutic modality to boost antitumor immunity.

## Introduction

Regulation of immune responses in the tumor microenvironment plays a vital role in cancer development and progression^1–5^. T cells in particular have demonstrated powerful antitumor potential that could be manipulated for cancer therapy. In animal models, CD8+ cytotoxic T cells are important for tumor inhibition by inducing malignant cell apoptosis in many types of cancers. Th1 polarized CD4+ T-helper cells are also important in orchestrating antitumor responses^6^. However, many tumors fail to recruit tumor-infiltrating CD8+ and Th1 CD4+ T cells^7–9^, resulting in an immunologically “cold” microenvironment that shields tumor cells from immune surveillance, thus allowing tumors to survive, proliferate, and metastasize. Additionally, therapeutic manipulations are often ineffective at eliciting effective antitumor T cell responses, suggesting that a critical barrier is established within the tumor microenvironment to suppress antitumor immune surveillance. However, the underlying mechanisms of immune evasion are not well understood.

The blood vasculature plays a critical role in tumors by regulating blood flow and immune cell recruitment^10^. Endothelial cells (ECs) lining the venular blood vessels are essential in governing T cell trafficking under physiological and pathological conditions^11, 12–14^. Molecular programs by which tumor ECs bind circulating leukocytes and mediate immune cell trafficking have been described^10,15,16^. However, the tumor vasculature often demonstrates disorganization, poor perfusion, and a compromised ability to recruit lymphocytes. These abnormalities are thought to be driven by aberrant or over-exuberant activation of angiogenic pathways in the tumor microenvironment.

Notch signaling, a key regulator of endothelial cell behavior, is commonly upregulated in the tumor endothelium^17–23^. While Notch ligands in the tumor tissue, including EC-derived Delta-Like 4 and tumor cell-derived Jagged 1, play well established roles in activating endothelial Notch^24,25^, the downstream consequences of Notch activation in ECs are not fully defined. In addition to regulating angiogenesis, Notch activation in blood endothelial cells drives the differentiation and maintenance of arterial endothelium while inhibiting veins and post-capillary venules^26,27^. Because post-capillary venules are the principal cites of leukocyte recruitment, we reasoned that abrogation of Notch signaling in ECs might enhance T cell homing into the tumors by activating venular programs. On the other hand, ECs are ubiquitous in the tumor tissue, and EC-derived angiocrine factors could engage other stromal cell populations in the microenvironment. We show here that selective abrogation of the canonical Notch pathway dramatically enhances T cell recruitment and activates tumor immunity, but not by altering vascular adhesion receptors for leukocyte homing. Instead, we found that endothelial cells promoted tumor immune evasion by suppressing the pro-inflammatory functions of cance-rassociated fibroblasts (CAFs).

As a crucial component of the tumor microenvironment, CAFs play diverse roles in tumor regulation, contributing to extracellular matrix deposition and modification, growth factor secretion, and immune modulation^28,29^. Studies on CAF depletion in animal models have yielded varied outcomes, suggesting both tumor-promoting and tumor-inhibiting functions of this cell type^30–34^. The paradoxical role of CAFs is thought to stem partly from the heterogeneity within the CAF compartment, as evidenced by single cell transcriptomic profiling that revealed the co-existence of CAF subsets, including myofibroblastic CAFs (myCAFs) and CAFs that express a large repertoire of cytokines (iCAFs)^35,36^. iCAF transcriptionally align with normal tissue-associated fibroblast subsets termed universal fibroblasts, which are thought capable of differentiating into myofibroblasts^37,38^. While distinct signaling pathways including TGFβ, IL1, and ERBB2 have been implicated in CAF subtype specialization^38–40^, the cellular drivers and the regulatory mechanisms governing CAF programming and functions remain incompletely understood.

Here we identify endothelial cells as a critical determinant of CAF functions in shaping the immunosuppressive tumor microenvironment in pancreatic ductal adenocarcinoma (PDAC). EC-specific abrogation of Rbpj, the master transcription factor of canonical Notch signaling, modulated the expression of angiocrine factors including TGFβ and PDGFβ, reprogrammed the CAF population from myofibroblasts into pro-inflammatory CAFs, and enhanced CAF-mediated recruitment of antitumor T cells via the CXCL9/10-CXCR3 pathway. Abrogation of Notch in ECs also unleashed interferon gamma responses in the tumor microenvironment, upregulated PDL1 expression in tumor cells, and sensitized PDAC to PD1-based immunotherapy.

## Results

### Notch signaling is upregulated in blood endothelial cells to promote PDAC growth.

**Figure 1.**
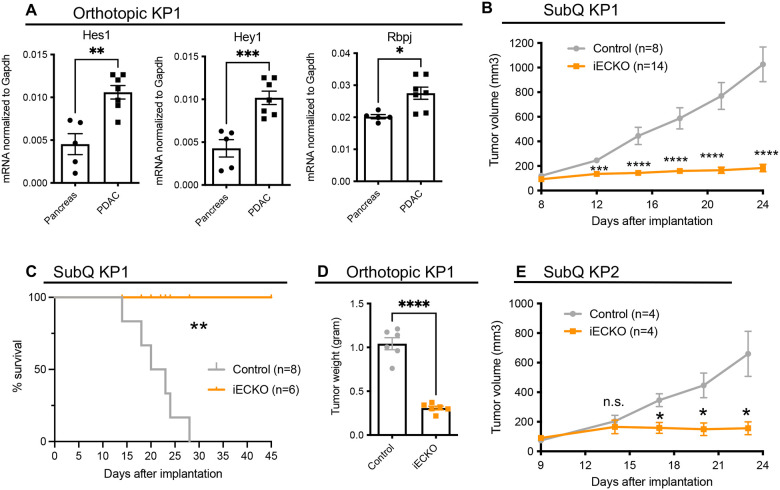
PDAC upregulates canonical Notch signaling in blood endothelial cells to promote tumor growth A. PCR quantification of Notch transcriptional target genes in blood endothelial cells from normal pancreas and orthotopic KP1 tumors. B. Volumetric measurements of subcutaneous KP1 tumors in control and Rbpj^iECKO^ mice. Representative of >5 independent experiments. (n=8–14/group) C. Survival of control and Rbpj^iECKO^ mice bearing subcutaneous KP1 tumors. (n=6–8/group) D. Weight measurement of orthotopic KP1 tumors in control and Rbpj^iECKO^ mice. Representative of >5 independent experiments. E. Volumetric measurements of subcutaneous KP2 tumors in control and Rbpj^iECKO^ mice. Representative of 3 independent experiments. (n=4/group) * p<0.05, ** p<0.01, *** p<0.001, **** p<0.0001 by student’s t-tests, except in Figure 1C analyses were performed by the Mantel Cox test. n.s. denotes not significant.

Human pancreatic ductal adenocarcinoma (PDAC) often displays limited T cell infiltration, which is recapitulated in mouse models^41,42^. To determine how blood vessels regulate immune cell recruitment and shape the tumor environment, we established orthotopic PDAC tumors using the KP1 cell line derived from autochthonous tumors from p48-Cre, Kras^G12D^, p53^f/f^ (KPPC) mice^42^. First, we asked whether Notch signaling is active in tumor blood endothelial cells (BECs). Towards this end, we isolated BECs (lineage-CD31+podoplanin-) from normal mouse pancreas and orthotopic KP1 tumors by fluorescence-activated cell sorting (**Figure S1A**), and performed quantitative polymerase chain reaction (QPCR) analyses to quantify the expression of Notch target genes. Compared to BECs isolated from normal mouse pancreas, the BECs in orthotopic tumors significantly elevated the expression of *Hey1*, *Hes1*, and *Rbpj* (Figure 1A), which are transcriptional targets activated upon engagement of canonical Notch pathways^43^. These data suggest that Notch signaling is upregulated in pancreatic BECs as the tumors develop.

To assess the role of endothelial Notch signaling in tumor growth, we genetically disrupted *Rbpj* in endothelial cells^44^ using the Cdh5(PAC)-Cre^ERT2^ × Rbpj^f/f^ mice (referred to as Rbpj^iECKO^ hereafter). *Rbpj* encodes an essential transcription factor that mediates canonical Notch signaling. We established subcutaneous tumors using the KP1 cell line, and treated Rbpj^iECKO^ mice with tamoxifen to delete Rbpj when tumors reached approximately 0.5cm in width. While tumors in control mice continued expanding in size after tamoxifen treatment, tumors in Rbpj^iECKO^ mice ceased growing shortly after the induction of Cre recombination (Figure 1B), at least doubling mouse survival duration (Figure 1C). Loss of Rbpj in ECs also significantly inhibited tumor growth in orthotopically implanted KP1 tumors (Figure 1D). Similar results were observed in subcutaneous and orthotopic tumors established using KP2 cells, an independent PDAC cell line derived from p48-Cre, Kras^G12D^, p53^m/wt^ (KPC) mice (Figure 1E). Collectively, these data suggest that canonical Notch signaling in tumor endothelial cells is necessary for tumor growth.

### Abrogation of endothelial Notch signaling promotes the infiltration of antitumor T cells, but not myeloid cells.

**Figure 2.**
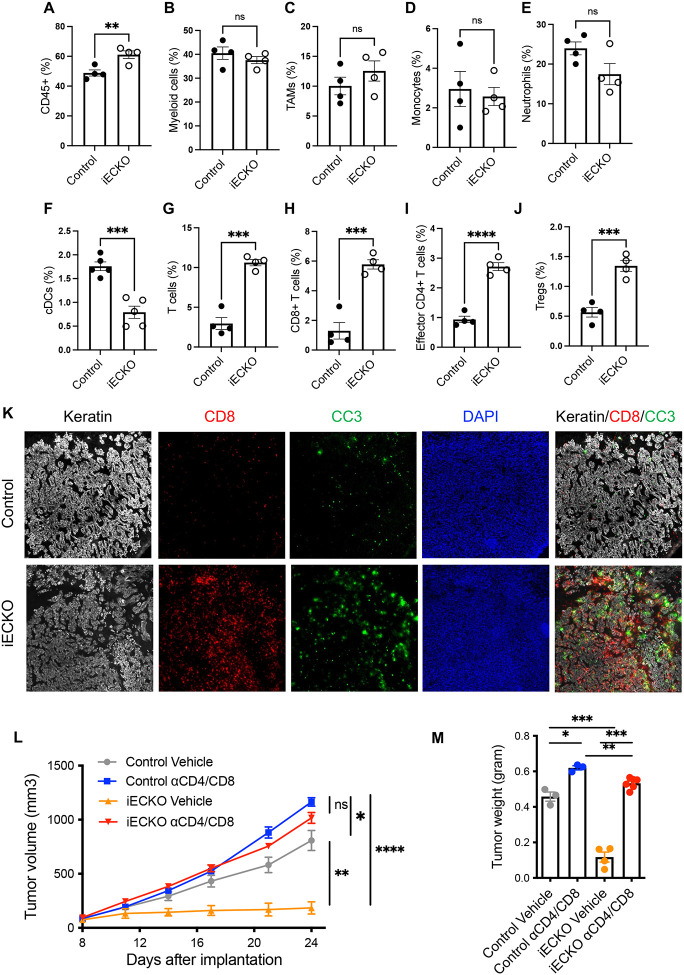
Abrogation of endothelial Notch signaling selectively promotes the infiltration of T cells in PDAC models. A-J. Flow cytometric quantification of indicated leukocyte populations in subcutaneous KP1 tumors of control and Rbpj^iECKO^ mice. Representative of >5 independent experiments. K. Immunofluorescence imaging of CD8 (red), cleaved caspase 3 (green), and keratin (white) in KP1 tumors from control and Rbpj^iECKO^ mice, visualized by the 10X objective. L-M. Volumetric (L) and weight (M) measurements of subcutaneous KP1 tumors in control and Rbpj^iECKO^ mice treated with CD4/CD8 depleting antibodies (n=3–6/group). Representative of >3 independent experiments. * p<0.05, ** p<0.01, *** p<0.001, **** p<0.0001 by student’s t-tests. Ns denotes not significant.

To determine whether canonical Notch signaling in endothelial cells regulates the immune landscape in the tumor tissue, we initially focused on immune cell recruitment. We deleted Rbpj in ECs of PDAC-tumor bearing mice, and profiled tumor-infiltrating leukocytes by flow cytometry. Abrogation of Notch signaling in ECs significantly increased the infiltration of total immune cells into the tumor (Figure 2A). However, Rbpj^iECKO^ did not significantly alter the abundance of tumor-associated macrophages (TAMs), monocytes/monocytic myeloid derived suppressor cells (Mo-MDSCs), neutrophils/granulocytic MDSCs (G-MDSCs), or eosinophils (Figures 2B–E, **S1B-D**). The abundance of tumor-infiltrating dendritic cells even decreased in Rbpj^iECKO^ mice (Figures 2F). On the other hand, loss of endothelial Notch signaling significantly increased the infiltration of T cells, including CD8+ and CD25-CD4+ effector T cells, and to a lesser extent regulatory T cells (T_reg_s) (Figure 2G–J), leading to an increased effector-to-Treg ratio. The upregulation in T cell infiltration occurred rapidly within 24 hours after tamoxifen induction before the manifestation of tumor burden differences (**Figure S2A**), suggesting that the increased T cell infiltration may be a driver of tumor inhibition. Immunofluorescence imaging confirmed the increased T cell infiltration into the tumor (Figure 2K). Interestingly, in PDAC tissues of Rbpj^iECKO^ mice, we observed clusters of CD8+ T cells surrounding cytokeratin-positive cells expressing cleaved caspase 3, which mark apoptotic tumor cells (Figure 2K). In independent orthotopic tumor models established using the KP2 cell line, we observed similar increases in the abundance of tumor-infiltrating T cells, but not of myeloid cells (**Figure S2B**). These data suggest that abrogation of Notch signaling in endothelial cells selectively promoted the infiltration of T cells in PDAC models.

To determine whether the increased T cell infiltration was necessary for tumor inhibition in mice with endothelial Notch deficiency, we treated tumor-bearing Rbpj^iECKO^ and control mice with CD4 and CD8 depleting antibodies. T cell depletion in the control mice only slightly increased tumor growth, suggesting that at baseline the antitumor activities of T cells were largely suppressed in this tumor model. Interestingly, while loss of Rbpj in ECs halted PDAC growth, depletion of T cells unleashed tumor progression to a level comparable to that seen in control mice (Figures 2L–M). Similarly, in orthotopic tumors, T cell depletion also abolished the Rbpj^iECKO^-mediated tumor inhibition (**Figure S2C-E**), indicating that enhanced infiltration of T cells was a necessary driver of Rbpj^iECKO^-mediated tumor inhibition.

Taken together, these findings indicate that the enhanced Notch signaling in tumor endothelial cells inhibits the infiltration of antitumor T cells, thereby shielding tumor cells from T cell-mediated killing. Abrogation of Notch signaling in ECs alleviated this inhibition and selectively enhanced T cell infiltration to halt cancer growth.

### Abrogation of Notch signaling in ECs reprograms cancer-associated fibroblasts.

**Figure 3.**
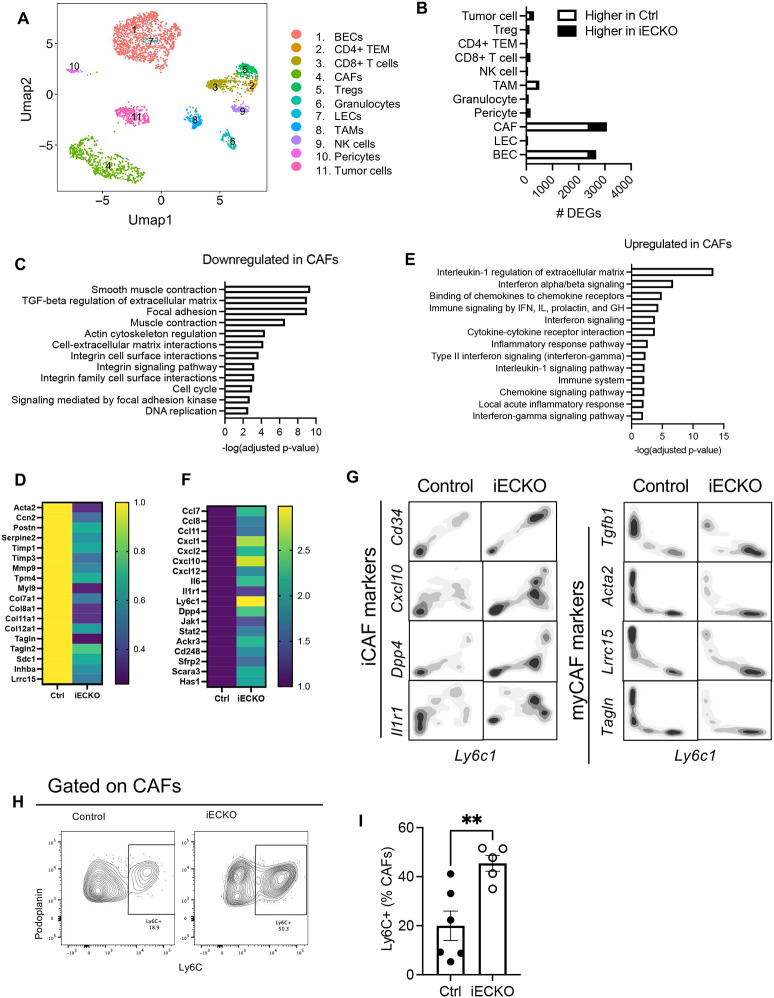
Abrogation of endothelial Notch signaling inhibits myofibroblastic CAFs and promotes pro-inflammatory CAF signature. A. Umap plots of tumor and stromal cells by scRNAseq analyses of PDAC in control and Rbpj^iECKO^ mice. B. Number of differentially expressed genes (DEGs) in indicated cell types in response to Rbpj^iECKO^, (p-value <0.01). C-D. BioPlanet pathway analyses (C) and heatmap (D) of DEGs in CAFs downregulated by Rbpj^iECKO^. E-F. BioPlanet pathway analyses (E) and heatmap (F) of DEGs in CAFs upregulated by Rbpj^iECKO^. G. Contour plot of scRNAseq of CAFs as marked by indicated molecules. H-I. Representative plots (H) and mean fluorescence intensity (MFI) quantification (I) of Ly6C expression in CAFs from KP1 tumors in control and Rbpj^iECKO^ mice. Representative of >5 independent experiments.* p<0.05 by student’s t-tests.

To identify mechanisms by which inhibition of endothelial Notch signaling activates T cell infiltration and antitumor immunity, we performed single cell transcriptomic profiling to understand how different cells change their programs in response to Notch abrogation in endothelial cells. To ensure sufficient representation of relevant cell types, we sorted ECs, tumor cells, T cells, cancer-associated fibroblasts (CAFs), natural killer (NK) cells, pericytes, and myeloid cells including TAMs, monocytes/Mo-MDSCs, neutrophils/G-MDSCs, and eosinophils (Figures 3A and **S1**). We treated mice with tamoxifen on Day 7 and Day 10 after tumor implantation, sorted cells on Day 11, and pooled the cells together for single cell RNA sequencing (scRNAseq). We selected this time point at which tumor burden differences were not manifest but the immune profile started to change (**Figure S2A**), with the goal of identifying the early drivers of Rbpj^iECKO^-mediated immune modulation.

As expected, abrogation of canonical Notch signaling significantly changed the gene expression profiles in blood endothelial cells, with 2421 genes downregulated and 246 genes upregulated by Rbpj deletion (p<0.01) (Figure 3B). In contrast, Rbpj deletion altered 67 genes in the lymphatic endothelial cells, which constituted only a minor fraction of ECs isolated from the tumor tissue. Therefore, we focused our analyses on BECs. We initially hypothesized that loss of Rbpj upregulated molecular programs of post-capillary venules to promote T cell recruitment. However, at our selected time point, we did not detect enhanced gene signatures associated with venular identity and functions. For example, we did not see increased expression of genes encoding molecules that promote leukocyte adhesion, including intercellular cell adhesion molecule-1 (*Icam1*), vascular cell adhesion molecule-1 (*Vcam1*), E-selectin (*Sele*), and P-selectin (*Selp*) (**Figure S3A-D**). We also did not see significantly upregulated expression of venular markers, including Darc (*Ackr1*), ephrinB4 (*Ephb4*), Neuropilin 2 (*Nrp2*), and COUP-TFII (*Nr2f2*), a master transcription factor for venular EC specification (**Figure S3E-H**). While we could not rule out the possibility of enhanced venular programs at later time points, these data suggest that Rbpj abrogation in ECs promoted rapid antitumor T cell immunity at early time points through other mechanisms.

Surprisingly, of all the cell types examined, CAFs had the largest number of genes differentially expressed between Rbpj^iECKO^ and control mice (Figure 3B), even exceeding the number of differentially expressed genes (DEGs) in ECs, with 2430 genes downregulated and 648 genes upregulated in CAFs of Rbpj^iECKO^ mice compared to their counterpart in control mice.

Before proceeding further with transcriptomic analyses, we performed lineage tracing studies to determine whether the CAFs were *de facto* fibroblasts or whether they constituted cells that transdifferentiated from ECs or tumor cells. Towards that end, we established KP1 tumors in Cdh5-Cre^ERT2^, Rbpj^loxP/loxP^ mice that carry a Rosa26^mTmG^ reporter, and deleted Rbpj by tamoxifen induction (**Figure S4A-B**). Fate mapping revealed that the majority (>90%) of the blood endothelial cells in the tumor express GFP, indicative of efficient Cre recombination in BECs (**Figure S4C-D**). However, we did not detect GFP signals in CAFs, suggesting that endothelial-to-mesenchymal (endoMT) transition did not significantly contribute to the composition of the CAF populations (**Figure S4C-D**). Moreover, tumor cells did not have a fluorescent reporter while most CAFs expressed tdTomato (**Figure S4E-F**), suggesting that CAFs did not derive from tumor cells undergoing epithelial-to-mesenchymal transition (EMT). Therefore, these data suggest that the CAF populations we assessed were *de facto* fibroblasts.

In response to the abrogation of Notch signaling in endothelial cells, CAFs downregulated a number of molecules involved in smooth muscle contraction, Rho-mediated motility, and extracellular matrix remodeling, including *Acta2, Tpm4, Myl9, Cfl1, Pfn1*, *Tpm6, Mylk, Myl6, Vcl, Lmod1*, and *Tln* (Figures 3C–3D), which suggest a loss of myofibroblast functions^35^. Additional genes associated with myofibroblast phenotypes, including *Ccn2 (Ctgf), Postn, Serpine2, Timp1, Timp3, Mmp9, Col7a1, Col8a1, Col11a1, Col12a1, Tagln, Sdc1, Inhba*, also decreased (Figure 3D). In addition, pathway analyses revealed a significant downregulation of TGFβ signaling, which is known to promote the myofibroblastic CAF (myCAF) phenotype^40^ (Figure 3C).

On the other hand, CAFs from Rbpj^iECKO^ mice upregulated genes associated with chemokine signaling, interferon signaling, and inflammatory responses (Figure 3E), with an increase in *Ccl7, Ccl8, Cxcl1, Cxcl2, Cxcl10, Cxcl12, Il6, Jak1, Stat2, and Ackr3* (Figure 3F). In addition, molecules indicative of inflammatory CAF (iCAF) phenotypes, including *Dpp4, Ly6c1, Cd248, Sfrp2, Scara3, and Has1*^45^, were significantly upregulated by Rbpj^iECKO^ as well (Figure 3F).

These shifts in gene expression correlated with an altered representation of CAF subsets upon the ablation of endothelial Notch signaling. scRNAseq revealed an enrichment of CAFs expressing iCAF markers, including *Ly6c*, *Cd34*, *Dpp4*, and *Cxcl10* in the Rbpj^iECKO^ mice (Figure 3G). Conversely, the fibroblast subset expressing myCAF markers, including *Lrrc15*, *Acta2*, and *Tgfb1*, was more abundant in the mice with intact Notch in ECs (Figure 3G). Consistent with the transcriptomic data, Rbpj^iECKO^ upregulated the representation of CAFs expressing Ly6C at the protein level (Figures 3H–I). Interestingly, one of the top pathways upregulated in CAFs was interleukin-1 (IL1) regulation of extracellular matrix (Figure 3E). Among the various tumor and stromal cell types, CAFs had the highest expression of Il1r1 (**Figure S5A**), the prototypical receptor that mediates IL1 signaling. CAFs also showed increased expression of *Il1r1* in Rbpj^iECKO^ mice (Figures 3F–G). These observations were consistent with previous reports that IL1 antagonizes TGFβ to promote the pro-inflammatory phenotypes and functions in CAFs^40^. Collectively the single cell profiling analyses suggest that abrogation of endothelial Notch reprogrammed the tumor fibroblast compartment from myofibroblastic CAFs into pro-inflammatory CAFs, which can bolster the engagement of immune responses^35^.

### Abrogation of endothelial Notch signaling alters angiocrine factors TGFβ and PDGFβ to reprogram CAFs.

**Figure 4.**
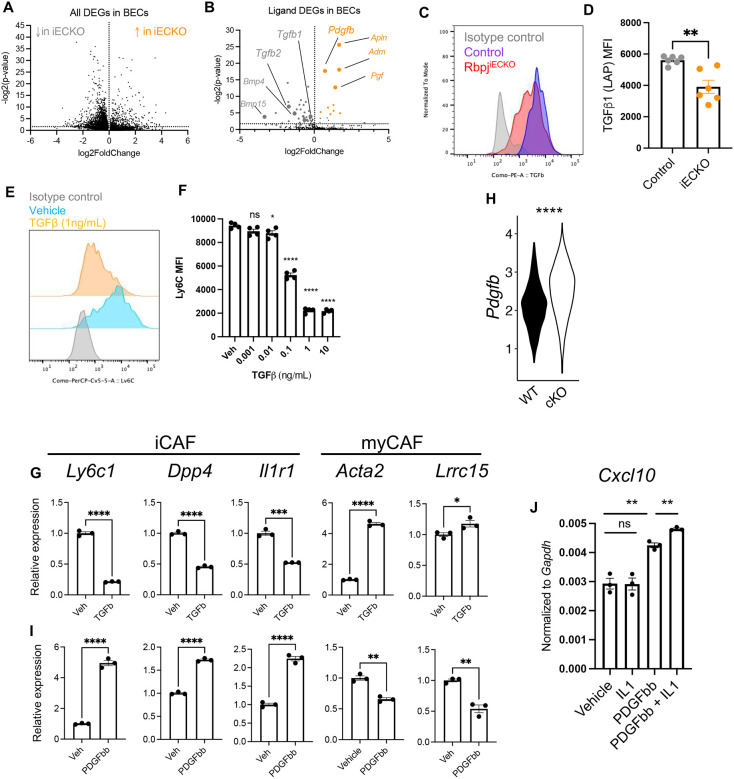
Abrogation of endothelial Notch modulates angiocrine PDGFβ and TGFβ to reprogram CAFs A. Volcano plot from scRNAseq analyses of differentially expressed genes (DEGs) in BECs of KP1 tumors in control and Rbpj^iECKO^ mice (p<0.01). B. Volcano plot of DEGs from (A) filtered by the GO term “ligand-receptor interactions”. C-D. Representative flow cytometric staining (C) and MFI quantification (D) of pro-TGFβ1 intracellular in BECs of orthotopic KP1 tumors from control and Rbpj^iECKO^ mice. E-F. Representative Ly6C staining (E) and Ly6C MFI quantification (F) on *ex vivo* CAFs treated with vehicle (PBS) or recombinant murine TGFβ at indicated doses. Analyses were done after 24 hours of culture. Representative of 3 independent experiments. G. QPCR analyses of iCAF and myCAF gene on *ex vivo* CAFs treated with vehicle (PBS) or recombinant murine TGFβ (1ng/mL). Data were normalized to the average of vehicle-treated samples. Representative of 3 independent experiments. H. Violin plot of *Pdgfb* expression in BECs from KP1 tumors of control and Rbpj^iECKO^ mice by scRNAseq. I. QPCR analyses of iCAF and myCAF gene on *ex vivo* CAFs treated with vehicle (PBS) or recombinant murine PDGFββ (10ng/mL). Representative of 3 independent experiments. J. QPCR analyses of Cxcl10 in CAFs treated with recombinant murine PDGFββ (10ng/mL) and/or IL1 (10ng/mL) for 24 hours. Representative of 3 independent experiments. * p<0.05, ** p<0.01, *** p<0.001, **** p<0.0001 by student’s t-tests. ns denotes not significant.

To understand how the loss of Notch signaling in ECs reprogrammed CAFs, we interrogated the differentially expressed genes in Rbpj-intact and -deficient BECs from KP tumors (Figure 4A). We hypothesized that interruption of Notch signaling changed the repertoire of EC-derived ligands, thus altering the signaling pathways downstream of the cognate receptors in CAFs. To address this hypothesis, we focused on DEGs in Rbpj-deficient vs. control BECs, and screened for genes whose ontology term falls into the “ligand receptor interactions” category. This filtered the DEG in BECs down to a list of 55 molecules (p<0.01) (Figure 4B, **Table S1**). Rbpj-deficiency in ECs downregulated the gene expression of 45 ligands, including *Tgfb1*, *Tgfb2*, *Bmp4, Bmp15*, *Kitl*, and *Pglyrp1*. Expression of 10 ligands was upregulated, including multiple molecules characteristic of angiogenic endothelium, such as *Apln*, *Adm*, *and Pgf*. Other upregulated ligand genes included *Gas6*, *Igf1*, and *Pdgfb*.

Consistent with the downregulation of the *Tgfb1* by scRNAseq (Figure 4B), the protein expression of pro-TGFβ in BECs was also reduced in response to Rbpj deletion (Figures 4C–D). TGFβ is a well-established regulator of fibroblast functions^46^ and is shown to drive myofibroblast specification in PDAC models^38,40^. To determine whether TGFβ could promote myCAF programming at the expense of iCAFs in our settings, we cultured primary fibroblasts isolated from orthotopic KP1 tumors, treated with varying doses of recombinant murine TGFβ, and assessed the expression of CAF marker Ly6C by flow cytometry. A dose of 0.1 ng/mL of TGFβ was sufficient to significantly decrease the protein expression of Ly6C (Figures 4E–F). QPCR analyses showed that TGFβ decreased the transcription of iCAF genes, including *Ly6c1*, *Dpp4*, and *Il1r1* (Figure 4G), while increasing the expression of myCAF markers, such as *Acta2* and *Lrrc15*, were upregulated by TGFβ (Figure 4G). These data suggest that abrogation of Notch signaling in BECs decreases the expression of TGFβ, thus dampening myofibroblastic CAF programming.

On the other hand, the expression of *Pdgfb* was upregulated in Rbpj^iECKO^ BECs (Figure 4H). While PDGFβ receptor is highly expressed in fibroblasts, its role in myCAF/iCAF programming has not been addressed. We treated CAFs *ex vivo* with recombinant murine PDGFββ. Contrasting with TGFβ, PDGFββ treatment enhanced the expression of multiple iCAF markers, including *Ly6c1* and *Dpp4*, but decreased the expression of myCAF genes, such as *Acta2* and *Lrrc15*. (Figure 4I). PDGFββ treatment also upregulated the expression of T cell recruitment chemokine *Cxcl10* in CAFs (Figure 4J). Moreover, PDGFββ significantly induced *Il1r1* expression in CAFs (Figure 4I), suggesting that PDGFββ may render CAFs more sensitive to IL1 signaling, thus synergistically facilitating iCAF programming. This was consistent with the upregulation of *Il1r1* expression and IL1 pathway in CAFs (Figure 3F). Indeed, while recombinant IL1 alone did not increase *Cxcl10* expression in cultured CAFs, PDGFβ activated IL1 responsiveness, resulting in synergistic upregulation of this chemokine (Figure 4J). Taken together, these data suggest that abrogation of endothelial Notch modulated the repertoire of EC-derived angiocrine factors, including PDGFβ and TGFβ, to drive iCAF reprogramming at the expense of myCAF specification.

### Reprogrammed CAFs, but not TAMs or G-MDSCs, are required for T cell-mediated antitumor immunity.

**Figure 5.**
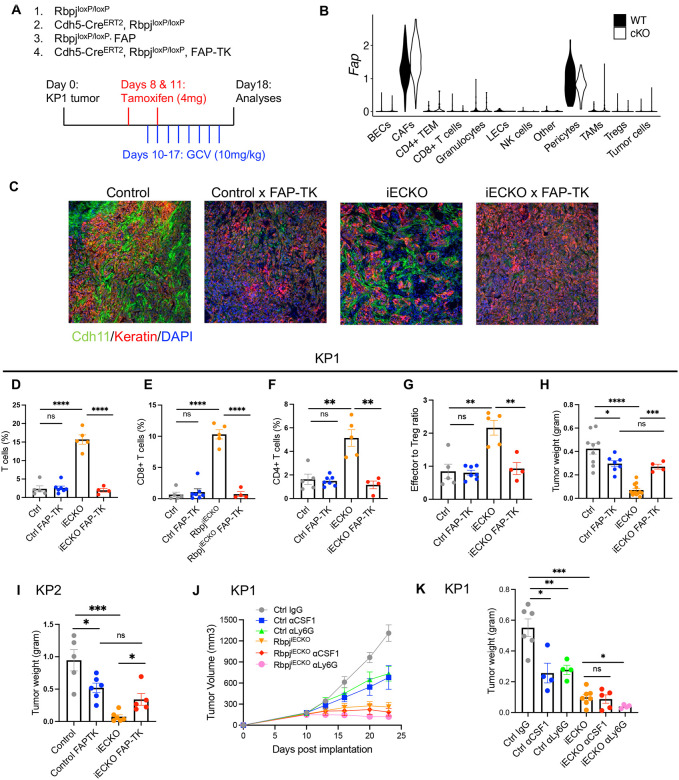
Reprogrammed CAFs, but not TAMs or G-MDSCs, are required for Rbpj^iECKO^-mediated T cell infiltration. A. Schematics of CAF depletion: KP1 or KP2 tumors were established in Rbpj^iECKO^ and control mice with or without the FAP-TK (fibroblast-activation protein-thymidine kinase), treated with tamoxifen to induce Rbpj manipulation, and injected with daily doses of ganciclovir (GCV) to deplete CAFs. B. Violin plots from scRNAseq analyses of *Fap* expression in indicated cell types from KP1 tumors from control and Rbpj^iECKO^ mice. C. Representative immunofluorescence imaging of CAFs and tumor cells, respectively stained by Cdh11 (green) and keratin (red), visualized by the 10X objective. D-G. Flow cytometric quantification of indicated T cell populations (D-F) and effector-to-Treg ratio (G) in KP1 tumors from FAP-depleted or FAP-intact Rbpj^iECKO^ and control mice. H. Wet tumor weight measurements of mice in (C-G). I. Weight measurements of orthotopic KP2 tumors in FAP-depleted or FAP-intact Rbpj^iECKO^ and control mice. J-K. Volumetric and weight measurements of subcutaneous KP1 tumors in control and Rbpj^iECKO^ mice. Mice were treated with tamoxifen on Day 10 and Day 13, followed by twice-weekly injections of aCSF1, aLy6G, or isotype control. * p<0.05, ** p<0.01, *** p<0.001, **** p<0.0001 by student’s t-tests. ns denotes not significant.

Next, we sought to determine whether CAFs were necessary for the changes in the tumor immune responses in Rbpj^iECKO^ mice. Towards this end, we depleted CAFs using a transgenic mouse model that expresses an inducible suicide gene, thymidine kinase (termed TK hereafter), driven by the fibroblast activation protein (FAP) promoter^33^ (Figure 5A). In the FAP-TK mice, administration of ganciclovir (GCV) induces the apoptosis of FAP-expressing cells^33^. In the PDAC models we used, FAP expression was restricted to cancer-associated fibroblasts and, to a lesser extent, in pericytes. Moreover, FAP was well expressed in most of the CAF populations of our tumor model (Figure 5B). We introduced the FAP-TK transgene into Rbpj^iECKO^ mice, which allowed us to selectively deplete CAFs and assess their functional importance in Rbpj^iECKO^-driven immune activation.

We implanted KP1 tumors into FAP-TK transgenic mice with or without Rbpj^iECKO^, treated mice with tamoxifen when tumors approached 0.5cm in width, and injected daily doses of ganciclovir before taking down the mice for analyses. FAP-TK efficiently depleted CAFs in both control and iECKO mice, as shown by the decreased immunofluorescence staining of Cdh11 (Figure 5C), which selectively marks CAFs (**Figure S5B**). When Notch was intact in ECs, CAF depletion did not significantly change the abundance of tumor-infiltrating T cells, consistent with previous reports demonstrating that FAP-depletion does not impact T cells in autochthonous PDAC^33^. These data suggest that CAFs were dispensable for the low level of T cell recruitment in the control mice. However, when Notch was abrogated in ECs, depletion of CAFs abolished the increase in tumor-infiltrating CD4+ and CD8+ T cells (Figure 5D–F). The Rbpj^iECKO^-driven increase in effector-to-Treg ratio was also reversed (Figure 5G).

Moreover, in mice with Notch-intact ECs, CAF depletion significantly decreased tumor burden, consistent with the previously reported tumor-supportive role of FAP+ CAFs in genetic PDAC models^33^. However, CAF depletion in Rbpj^iECKO^ mice reversed the tumor burden to a level comparable to that seen in FAP-TK mice with intact Rbpj in ECs (Figure 5H). In other words, upon CAF depletion, Notch abrogation in endothelial cells did not inhibit tumor growth. In orthotopic KP2 tumors, we also saw the same impact of CAF depletion on tumor burden (Figure 5I), suggesting that when Notch was abrogated, tumor-promoting CAFs were reprogrammed to inhibit tumor growth. Taken together, these data suggest that CAF reprogramming was an integral part of the stromal remodeling that induces antitumor immunity in response to the abrogation of endothelial Notch.

Because of the abundance and the immunomodulatory roles of TAMs and neutrophils/G-MDSCs in PDAC, we also asked whether these myeloid cells participated in Rbpj^iECKO^-driven tumor inhibition. To deplete these cells, we treated KP1 tumor-bearing mice with antibodies against CSF1 and Ly6G respectively. Both TAM and neutrophil depletion reduced tumor burden in mice that had intact endothelial Notch, confirming the previously reported tumor-promoting functions of these myeloid cells^47,48^. In Rbpj^iECKO^ mice, TAM depletion did not affect tumor growth, and neutrophil depletion slightly decreased tumor burden (Figure 5J–K), suggesting that neither of these myeloid populations was necessary for tumor restraint seen in Rbpj^iECKO^ mice. These data, along with studies using the FAP-TK mouse model, underscore the importance of CAF reprogramming in EC regulation of antitumor immunity.

### Abrogation of endothelial Notch signaling enhances CXCR3-mediated recruitment of antitumor T cells through CAF-derived CXCL9 and CXCL10.

**Figure 6.**
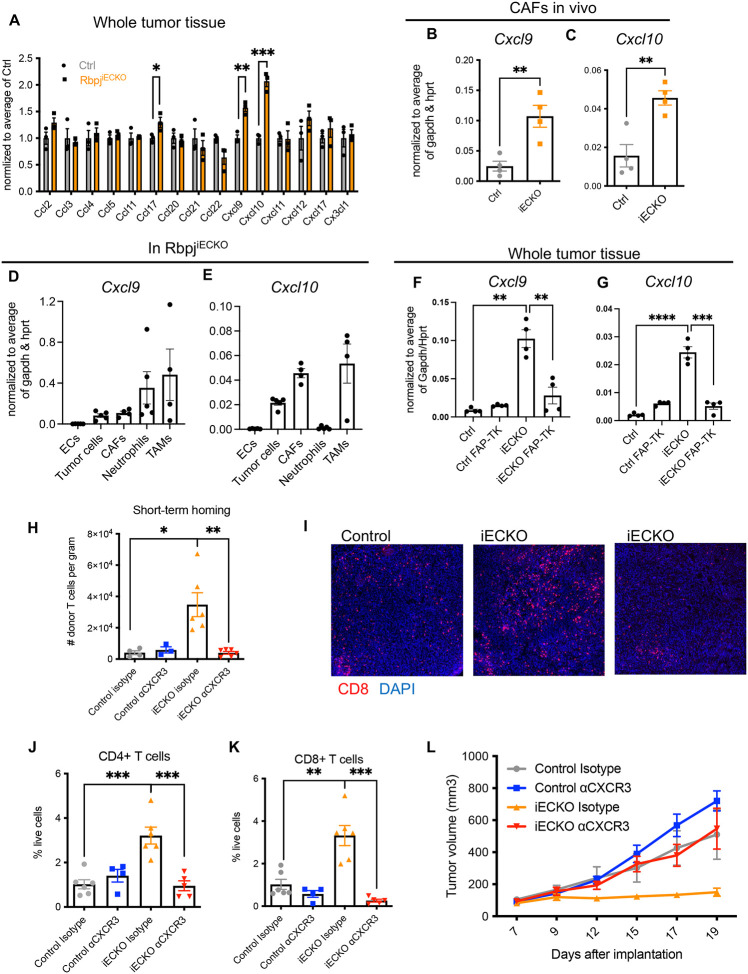
Abrogation of endothelial Notch signaling enhances CXCL10/CXCR3-mediated recruitment of antitumor T cells. A. QPCR quantification of chemokine array on whole tissues of KP1 tumors in control and Rbpj^iECKO^ mice. B-E. QPCR analyses of *Cxcl9* and *Cxcl10* in CAFs (B-C) and other cells (D-E) isolated from orthotopic KP1 tumors in control and Rbpj^iECKO^ mice. F-G. QPCR analyses of *Cxcl9* and *Cxcl10* in whole tissues of subcutaneous KP1 tumors in control and Rbpj^iECKO^ mice with FAP-expressing cells intact or depleted. H. Quantification of donor-derived T cells recruited to orthotopic KP1 tumors in control and Rbpj^iECKO^ recipient mice treated with anti-CXCR3 or isotype control. I. Immunofluorescence imaging of CD8+ T cells in KP1 tumors in control and Rbpj^iECKO^ mice, visualized by the 10X objective. J-K. Flow cytometric quantification of CD4+ and CD8+ T cells on tissues in (I). L. Volume measurement of tumors in (I-K). Representative of 3 independent experiments. * p<0.05, ** p<0.01, *** p<0.001, **** p<0.0001 by student’s t-tests. ns denotes not significant.

We next investigated how the EC-CAF remodeling enhanced T cell recruitment. Given the observations that the infiltration of T cells, but not the myeloid cells, was enhanced in Rbpj^iECKO^ tumors, we hypothesized that loss of endothelial Notch signaling led to increased production of chemokines in the tumor stroma to promote T cell recruitment. To identify the chemokines, we isolated mRNA from KP1 tumor tissues established in Rbpj^iECKO^ and control mice, and profiled the expression of an array of chemokines by QPCR analyses. We did not see increased expressions of most chemokines that we examined, including *Ccl2*, *Ccl3*, *Ccl5*, *Ccl11*, and *Cx3cl1* (Figure 6A). These were consistent with the observations that Notch deficiency in ECs did not alter the infiltration of myeloid cells, including monocytes/TAMs and eosinophils. On the other hand, the expression of *Cxcl9* and *Cxcl10* significantly increased in the tumors of Rbpj^iECKO^ mice (Figure 6A). CXCL9 and CXCL10 mediate T cell recruitment through the receptor CXCR3, which is predominantly expressed on activated CD8+ T cells and Th1 differentiated CD4+ T cells^49–51^. We thus hypothesized that abrogation of endothelial Notch promotes T cell recruitment via the CXCL9/10-CXCR3 pathway.

Because CAFs were indispensable for Rbpj^iECKO^-mediated T cell recruitment, we hypothesized that CAFs drove the increased expression of CXCL9 and CXCL10. To address this possibility, we sorted CAFs and quantified the mRNA expression of these chemokines. Compared to cells sorted from control mice, CAFs in Rbpj^iECKO^ mice had significantly increased expression of the *Cxcl9* and *Cxcl10* transcripts (Figure 6B–C). By contrast, the expression of both chemokines was not significantly changed by Rbpj^iECKO^ in other cell types that we assessed, including BECs, tumor cells, neutrophils, and TAMs (**Figure S6**). TAMs and neutrophils/G-MDSCs were the highest expressers of *Cxcl9*, followed by CAFs and tumor cells (Figure 6D). On the other hand, CAFs and TAMs expressed the highest amount of *Cxcl10* in Rbpj^iECKO^ mice (Figure 6E). ECs were not significant sources of CXCL9/10 (Figures 6D–E). Importantly, FAP-TK-mediated depletion of CAFs in Rbpj^iECKO^ mice abolished the increase in the expression of *Cxcl9* and *Cxcl10* in the whole tumor tissue homogenates (Figure 6F–G), suggesting that reprogrammed CAFs are important sources of CXCL9 and CXCL10 upregulation in the tumor microenvironment.

To determine whether the CXCL9/10-CXCR3 signaling was required for enhanced T cell recruitment, we assessed the impact of CXCR3 blockade on short-term lymphocyte homing (Figure 6H). We established orthotopic KP2 tumors in Rbpj^iECKO^ and control recipient mice, and treated the mice with tamoxifen when the tumors became palpable. For donors we established orthotopic KP2 tumors in Rosa26-mTmG mice, whose cells carry tdTomato fluorescence. We then isolated lymphocytes from the tumor-draining lymph nodes (LNs) of these donor mice and injected them intravenously into the recipient mice. Concomitantly with cell transfer, mice were treated intraperitoneally with the CXCR3 blocking antibody or the isotype control. Sixteen hours after injection, we dissociated tumors from the recipient mice and performed flow cytometry to quantify the number of donor-derived cells (tdTomato+) that have homed to the tumor tissue. Consistent with our hypothesis, Rbpj^iECKO^ significantly increased the tumor homing of donor-derived T cells; and this increase was reversed upon CXCR3 blockade. These data suggest that CXCR3 mediated the enhanced recruitment of T cells into PDAC tumors in Rbpj^iECKO^ mice (Figure 6H).

To determine whether the CXCL9/10-CXCR3 pathway was required for Rbpj^iECKO^-mediated tumor inhibition, we treated KP1 tumor-bearing mice with CXCR3 blocking antibody or isotype control. Anti-CXCR3 treatment successfully blocked the increase in the infiltration of CD8+ and CD4+ T cells into the tumor tissue in Rbpj^iECKO^ mice, without altering the baseline abundance of T cells in control tumors with intact endothelial Notch (Figures 6I–K). Importantly, CXCR3 blockade abolished the inhibition of tumor growth seen in Rbpj^iECKO^ mice (Figure 6L), suggesting that the tumor inhibition induced by Notch deficiency in ECs depends on CXCR3-mediated T cell infiltration. These observations were most likely directly due to the blockade of CXCR3 on T cells, because CXCR3 was expressed predominantly on T cells, but not on tumor cells, ECs, or other stromal populations in this tumor model (**Figure S7**). Taken together, these data suggest that endothelial Notch signaling blocks CXCL9/10-CXCR3-mediated T cell homing to shape a microenvironment that shields tumor cells from immune surveillance. Upon the abrogation of Notch in the endothelium, CAFs are reprogrammed from a myofibroblast-dominated compartment into an iCAF-enriched population, releasing CXCL9/10 production to promote T cell recruitment via CXCR3.

### Abrogation of Notch signaling in ECs unleashed interferon gamma responses in the tumor microenvironment and sensitized tumor responses to PD1 blockade.

**Figure 7.**
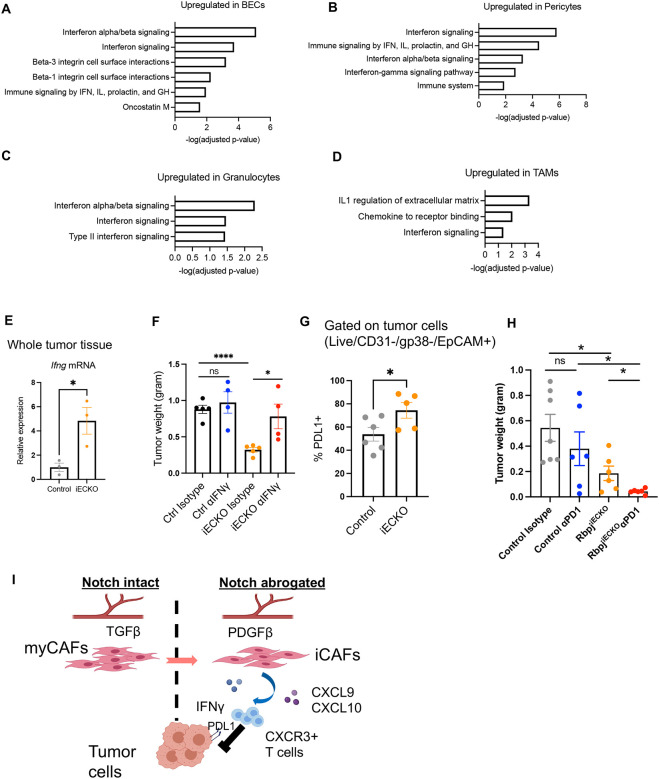
Abrogation of endothelial Notch unleashes IFNγ responses and sensitizes PDAC to PD1 blockade. A-D. Select BioPlanet pathway analyses of indicated tumor stromal populations that are upregulated by Rbpj^iECKO^. (IFN: interferon, IL: interleukin, GH: growth hormones) E. QPCR quantification of *Ifng* transcript in KP1 whole tissue lysates of control and Rbpj^iECKO^ mice. F. Weight measurements of orthotopic KP1 tumors in Rbpj^iECKO^ mice treated with αIFNγ or isotype control. Representative of 2 independent experiments. G. Flow cytometric analyses of PDL1 expression in KP1 tumor cells from control and Rbpj^iECKO^ mice. Representative of 2 independent experiments. H. Weight measurements of orthotopic KP1 tumors in Rbpj^iECKO^ mice treated with αPD1 or isotype control. I. Proposed model of Rbpj^iECKO^-driven T cell responses via CAF remodeling. * p<0.05, **** p<0.0001 by student’s t-tests. ns denotes not significant.

scRNAseq revealed a robust Rbpj^ECKO^-dependent upregulation of genes associated with type II interferon signaling in a wide spectrum of stromal cells, including not only CAFs, but also BECs, pericytes, TAMs, and granulocytes (Figures 7A–D). Consistent with scRNAseq data, the expression of interferon gamma (IFNγ) transcripts in the tumor tissue significantly increased in Rbpj^iECKO^ mice (Figure 7E). Neutralization of IFNγ reversed the suppression of tumor growth in Rbpj^iECKO^ PDAC-bearing mice, suggesting that the interferon responses were important for the Rbpj^iECKO^-mediated tumor inhibition (Figure 7F).

The increased IFNγ expression in Rbpj^iECKO^ mice correlated with increased PDL1 expression on tumor cells (Figure 7G), consistent with the known ability of IFNγ to induce the transcription of PDL1^52^. Therefore, even though endothelial deficiency in Notch enhances T cell recruitment, engagement of PDL1 could help tumor cells evade surveillance from antitumor T cells. On the other hand, we hypothesized that this upregulation could sensitize PDAC to PD1-based immunotherapy. To address this hypothesis, we injected Rbpj^iECKO^ and control mice with orthotopic KP1 tumors, induced Rbpj deletion with tamoxifen at a late time point when tumors reached approximately 1cm in diameter, and then treated mice with PD1 antagonist antibody or isotype control. PD1 blocking antibodies alone in control mice did not significantly reduce tumor weight, recapitulating the resistance to immune checkpoint blockade seen in human PDAC patients. However, in tumor-bearing Rbpj^iECKO^ mice with PD1 blocking antibodies decreased tumor burden markedly by 78% (Figure 7H). Taken together, these data suggest that loss of Notch signaling in tumor ECs unleashes interferon gamma responses in the tumor microenvironment, upregulates PDL1 expression in tumor cells, and sensitizes tumors to PD1-based immunotherapy.

## Discussion

In this study we show that blood endothelial cells in PDAC upregulated canonical Notch signaling to inhibit the pro-inflammatory functions of cancer-associated fibroblasts, thereby protecting tumors from T cell-mediated attacks. Abrogation of Notch signaling in ECs altered endothelial expression of angiocrine factors including TGFβ and PDGFβ, reprogrammed myofibroblastic CAFs into pro-inflammatory CAFs, and promoted the recruitment of antitumor T cells into the tumor tissue via the CXCL9/10-CXCR3 pathway. This EC-CAF remodeling also unleashed Type II interferon responses in the tumor microenvironment, upregulated PDL1 expression on tumor cells, and sensitized tumors to PD1-based immunotherapy (Figure 7I).

Emerging evidence suggests that the vasculature engages multiple mechanisms to inhibit effector T cell infiltration for tumor immune evasion. Tumor-associated blood endothelial cells can induce apoptosis in incoming T cells in a Fas-dependent manner^53^. Tumors can also inhibit the ability of blood endothelial cells to recruit T cells. For example, BECs downregulate the expression intercellular adhesion molecule-1 (ICAM-1), reducing lymphocyte adhesion to the tumor vessels^54^. In an independent study, we also saw reduced frequencies of post-capillary venules expressing leukocyte adhesion molecules during tumor expansion, providing another mechanism of EC-orchestrated T cell scarcity. Here we discovered that ECs, through Notch-regulated angiocrine factors, exert pronounced effects on the tumor stroma to inhibit T cell recruitment chemokines CXCL9/10 and promote immune evasion.

CXCL9/10 and their receptor CXCR3 are important mediators of Th1 CD4+ and effector CD8+ T cell trafficking in animal models of inflammation and cancer^55,56^ and may play comparable roles in cancer patients. In PDAC patients, higher plasma concentrations of CXCL9/10 correlate with improved overall survival^57^. An abundance of tumor-infiltrating CXCR3+ T cells predicts favorable PDAC responses to FOLFIRINOX^58^. In the KPC models used here and with intact endothelial Notch, CXCR3 blockade did not substantially change T cell infiltration or tumor size, suggesting that CXCR3-mediated T cell recruitment is not significantly engaged. However, the power of CXCL9/10 to recruit CXCR3+ antitumor T cells was reactivated upon endothelial Notch deletion, elevating IFNγ in the tumor microenvironment. IFNγ is a potent activator of CXCL9/10, and IFNγ treatment significantly induced *Cxcl9/10* expression in CAFs *ex vivo* (data not shown), raising the possibility that IFNγ-producing T cells could self-amplify CAF-mediated antitumor immunity. It is worth noting that CXCR3 can also orchestrate the intratumor spatial localization of infiltrated T cells, thus subverting the “immune excluded” environment to augment antitumor immune responses^59^.

Our results reveal an unexpected role of endothelial cells in the regulation of cancer-associated fibroblast functions. A growing body of studies highlights the role of cancer-associated fibroblasts in immune modulation^28,38,60,61^. CAFs are potent producers of cytokines and chemokines that recruit TAMs, MDSCs, and regulatory T cells, which collectively suppress T cell functions^60,62–65^. CAFs can also create a microenvironment rich in extracellular matrix molecules, establishing a fibrotic barrier to physically shield T cells from accessing tumor cells. Additionally, CAFs can also directly suppress CD8+ T cell proliferation and induce T cell exhaustion and apoptosis^66,67^, further promoting tumor immune evasion. Unexpectedly, our results identified endothelial cells and EC-intrinsic Notch signaling as an upstream determinant of CAF phenotypes and functions, and showed that the CAF compartment that facilitates tumor progression can be reprogrammed to be an indispensable driver of antitumor T cell immunity.

Mechanistically, our data suggest that ECs regulate CAF functions through Rbpj-modulated angiocrine factors. Genes for 55 endothelial-derived ligand genes were altered when Rbpj was deleted in ECs. The most prominent change was the downregulation of TGFβ1, a prototypical cytokine known to drive myofibroblastic CAF specification^40^. Notably, multiple genes in the TGFβ superfamily were downregulated by Rbpj deletion. The other members, including TGFβ2 and BMP2, have also been implicated in fibroblast functions in other disease settings^68,69^ and may contribute to EC modulation of CAFs. Genes upregulated by Rbpj deletion also suggest mechanisms by which ECs can modulate CAFs. We focused on PDGFβ, a cytokine known for fibroblast/pericyte recruitment^70^. Unexpectedly, recombinant PDGFββ not only promoted iCAF marker expression *ex vivo*, but also sensitized fibroblasts to IL1-mediated iCAF programming. Despite the increased *Pdgfb* expression in BECs, we did not see a dramatic transcriptional reprogramming of pericytes in scRNAseq. Other upregulated molecules, such as IGF1, may also participate in fibroblast remodeling. Further analyses of the Notch-regulated EC secretome may provide additional insights into the regulation of CAFs by the tumor endothelium. Despite the net effects of tumor inhibition, it is important to note that Rbpj deletion alters the expression of both pro- and anti-tumor cytokines in ECs in a complex manner, not in all cases correlating with the effect on tumor growth. The complexity of the local angiocrine niche, the timing of angiocrine secretion and other factors within the tumor microenvironment may integrate to coordinate the anti-tumor responses after the abrogation of Notch signaling in ECs.

We correlate Rbpj^iECKO^-induced tumor inhibition with enhanced effector T cell recruitment, suggesting that stromal regulation of immune cell recruitment is a core mechanism not only for reduced leukocyte access, but also for active tumor immune evasion. Whether this local mechanism is sufficient to explain the activation of the immune response remains to be determined. Pan-endothelial genetic manipulation affects not only the tumor blood vasculature but also lymphatic vessels and vessels in distant sites, such as the tumor draining lymph nodes. Such perturbations could also contribute to the effects of endothelial Notch signaling on the immune system, modulating the potent effects of BEC reprogramming we describe here.

Here we focused on EC-intrinsic Notch signaling through the canonical Rbpj-mediate pathway. Notch signaling in the tumor microenvironment is complex, and Notch targeting by different modalities (e.g. anti-DLL4 and gamma secretase inhibitors) results in mixed outcomes in different tumor contexts^71^. Notch regulates angiogenesis and cancer stem cell maintenance in addition to tumor immune evasion^24,72^, and Notch targeting strategies must consider these complex interactions and effects. The ubiquity of this pathway in various cell types underscores the necessity of a nuanced understanding of its role in specific cell types and highlights the need for cell type-specific targeted delivery methods. Among tumor stromal cells, the blood vascular endothelium is uniquely accessible to such approaches, which can be delivered via intravascular routes. Indeed, recent advances in nanoparticle delivery systems targeting EC-specific gene expression and deletion suggest the potential for clinical applications of tumor endothelial-selective gene modulation in the future^73^.

In summary, we describe a pivotal role of endothelial cell Notch signaling in remodeling the tumor stroma and promoting immune evasion. Our studies highlight the importance of EC-CAF interactions in sculpting the tumor immune microenvironment and suggest the targeting of EC-CAF interactions as a therapeutic strategy to boost antitumor immunity.

## METHODS

### PDAC Model and Treatment

To establish KPC models, between 20,000 and 100,000 KP1 or KP2 cells in 50 μL of Cultrex (Trevigen) were injected orthotopically into the pancreas or subcutaneously into the left flank of 2- to 6-month-old mice. Once tumors became palpable orthotopically or reached 0.5cm in length subcutaneously, Cdh5-Cre^ERT2^; Rbpj^loxP/loxP^ and control mice (Cdh5-Cre^ERT2^ only or Rbpj^loxP/loxP^ only) were treated with two doses of 150ug/kg tamoxifen. Concurrent with the first tamoxifen injection, we also started treating mice intraperitoneally (i.p.) with antibodies (BioXCell) against CXCR3 (CXCR3–173, 10mg/kg), IFNγ (XMG1.2, 200mg), CD4 (GK1.5, 200mg), CD8β (53–5.8, 200mg), or isotype controls, including Armenian hamster IgG and rat IgG1 (HRPN) or IgG2b (LTF-2), twice a week. FAP-TK and control mice were injected intraperitoneally with daily doses of ganciclovir (Invivogen) at 10mg/kg starting at indicated time points. The volume of subcutaneous tumors was quantified as ½ × length × width^2^. Survival was scored when mice lost 15% of body weight, tumors reached 1.5cm in diameter, or upon death. All animal work was approved by Institutional Animal Care and Use Committee at the Veterans Affairs Palo Alto Health Care System.

### Tissue Isolation and Flow Cytometry

Tumor tissues were manually minced and digested in 20 mL of Hanks Balanced Salt Solution (HBSS) (Thermo Fisher) containing 500U/mL collagenase D (Worthington Biochem), 20 μg/mL DNase I (Sigma), and 2% fetal bovine serum (FBS) for 30 min at 37C with constant stirring. Normal pancreas was digested for 15 min. Digestion was quenched by ethylenediaminetetraacetic acid and filtered through 40 μm Nylon mesh, pelleted through centrifugation (750g for 5 min at 4C), and resuspended in phosphate buffered saline (PBS).

Single cell suspensions were incubated in PBS with anti-mouse CD16/CD32 antibodies (1/200) and Zombie NIR^™^ Fixable Viability dye (1/500) (BioLegend) for 10 min, pelleted by centrifugation, and subsequently labeled with 100–200 μL of fluorophore-conjugated anti-mouse antibodies at pre-determined dilutions for 20 min on ice, and washed with staining buffer (PBS with 1% FBS). Cells were fixed in 4% formaldehyde for 30 minutes, and washed in staining buffer. For pro-TGFβ staining, cells were stained for surface antigens, fixed and permeabilized using the eBioscience^™^ FoxP3/Transcription Factor Staining Buffer Set (Thermo Fisher), and then incubated with the TGFβ antibody. For proliferation assays, mice were injected with BrdU (4 mg) i.p. 16 hr prior to sacrifice. BD Bioscience Cytofix/cytoperm kit was used to stain for BrdU. Data were acquired on LSR-Fortessa (BD Biosciences) and analyzed using FlowJo software.

### Lymphocyte Homing Assay

We implanted 50,000 KP1 tumor cells orthotopically in the pancreas of donor (Cre^Neg^, Rosa26-mTmG) and recipient (Cdh5-Cre^ERT2^; Rbpj^loxP/loxP^ and control) mice. When tumors reached approximately 1cm in diameter, we injected recipient mice with tamoxifen (4mg/dose) on two consecutive days. Twenty-four hours later, we isolated the hepatic, portal, and mesenteric lymph nodes of donor mice, crushed through 40 filters, treated with red cell lysis buffer (BioLegend), washed and resuspended in 200 μL of PBS, and injected intravenously into the recipient mice. Approximately 10^7^ live cells were injected into each recipient. Donor cells were confirmed to be tdTomato+. Sixteen hours after injection, we sacrificed recipient mice for flow cytometry analyses.

### RNA Isolation and Quantitative PCR

RNA was isolated using E.Z.N.A. Total RNA kit (Promega) from snap frozen tissues or from sorted live cells. cDNA was synthesized using qScript cDNA SuperMix (Quantabio) following manufacturer instructions. Quantitative PCR was performed using SYBR Green Master mix (Thermo Fisher). Primers were either designed using Primer3 or referenced from PrimerBank and synthesized by Integrated DNA Technologies.

### Immunofluorescence Imaging

Fresh tumor tissues were fixed in 2% formaldehyde overnight, incubated with 30% sucrose overnight, and embedded in OCT compound (Sakura Finetek). Six μm cryosections were blocked with PBS and 5% serum (1hr at room temperature), incubated with rabbit anti-cleaved caspase 3 (Cell Signaling) or anti-Cdh11 (LS Bio) overnight, and then with Alexa488-conjugated goat anti-rabbit IgG (ThermoFisher), PE-CD8 (BioLegend), and Alexa594- or Alexa647-conjugated anti-pan-keratin (C11, Cell Signaling) antibodies for 1hr at room temperature. Tissues were washed with PBS containing 0.05% Tween-20 between incubation steps, and mounted in DAPI-containing Fluoromount-G (SouthernBiotech) for imaging on Apotome (Zeiss).

### Single-cell RNA sequencing

KP1 tumor cells (100,000) were established in Rbpj^iECKO^ and control mice (8–9 mice/group). Mice were treated with 150mg/kg of tamoxifen on Day 10 and 13, and sacrificed on Day 14. Cells from Rbpj^iECKO^ and control mice were isolated in parallel and stained with TotalSeq-A hashtag antibodies (BioLegend) simultaneously with flow antibodies. Populations of interest were then sorted and processed for scRNAseq using Chromium Single Cell 3’ Library and Gel Bead Kit v3.1 (10x Genomics) according to manufacturer guidelines. Male and female mice from Rbpj^iECKO^ or control cohort were processed together and resolved post-sequencing. Libraries were sequenced on NovaSeq 6000 (Illumina) at Stanford Functional Genomics Facility. Cell Ranger (v6.0.1, 10x Genomics) was used to align reads to the mm10 reference genome and perform quality control. Count data were processed with the Seurat package (v4.0.2). Raw counts were log normalized and 2000 most variable genes were identified based on a variance stabilizing transformation. Principal Component Analysis (PCA) dimensionality reduction was performed using the variable gene sets. Cell clusters were determined using a Shared Nearest Neighbor (SNN) modularity optimization-based clustering algorithm of the Seurat “FindClusters” function, and refined manually based on subset marker expression. To recover gene-gene relationships that are lost due to dropouts, missing gene expression data from log normalized count data was imputed using the MAGIC (Markov Affinity-based Graph Imputation of Cells) algorithm with optimized parameters (*t* = 2, *k* = 9, *ka* = 3)^74^. Imputed data were used for visualization of single-cell gene expression in violin plots. Differential gene expression analysis was performed using the “FindMarkers” function of the Seurat package on log normalized count data (p<0.05). Gene enrichment analysis of DEGs with BioPlanet databases was performed using Enrichr^75^.

### Quantification and Statistical Analysis

Statistical analysis was performed in Prism 10 (GraphPad Software) or R studio. Statistical significance was evaluated by Student’s t test or Mantel-Cox test, unless otherwise noted. *p < 0.05, **p < 0.005, ***p < 0.0005, ****p < 0.0001. n.s. denotes not significant. n represents number of replicates.
